# Supplementary material for: Resistance to different anthracycline chemotherapeutics elicits distinct and actionable primary metabolic dependencies in breast cancer
Source: eLife. 2021 Jun 28;10:e65150. doi: 10.7554/eLife.65150 (PMC8238502; doi:10.7554/eLife.65150)
Supplement: Supplementary file 1. [file elife-65150-supp1.docx]

**Supplementary File 1: PGC-1α supports therapeutic resistance across several cancer types**

| **Cancer type** | **Drug** | **PGC-1α role in resistance** | **Mechanism** | **Reference** |
| --- | --- | --- | --- | --- |
| Non-Small Cell Lung Cancer | Cisplatin | Promotes | Resistant cells overexpress PGC-1α and PGC-1β to promote mitochondrial biogenesis, thereby overcoming reduced OXPHOS inefficiency caused by mtDNA mutations | (1) |
| Ovarian cancer | Cisplatin | Promotes | Resistant cells overexpress PGC-1α and are dependent on OXPHOS | (2) |
| Ovarian cancer | Cisplatin or Paclitaxel | Promotes | Treatment increases ROS, which increases PGC-1α and reprograms metabolism | (3) |
| Breast cancer | Metformin | Promotes | Metformin-resistant cells show elevated PGC-1α and high-PGC-1α tumors are resistant to metformin | (4) |
| Colon cancer | Oxaliplatin and 5-FU | Promotes | Chemotherapy induces a SIRT1/PGC1α-dependent increase in OXPHOS that promotes tumor survival during treatment | (5) |
| Breast cancer | PI3K/mTOR inhibitors | Promotes | PGC-1α and ERRα promote lactate oxidation, which is a pathway preferred by resistant cells | (6) |
| Melanoma | Piperlongumine | Promotes | Subset of tumors show high PGC1α, increased mitochondrial capacity and resistance to oxidative stress | (7) |
| Glioblastoma stem cells | Radiation | Promotes | Resistant cells overexpress PGC-1α | (8) |
| Melanoma | Selumetenib | Promotes | Subset of resistant melanomas show high PGC1α and OXPHOS; mTORC1/2 inhibitors are effective against this subset. | (9) |
| Ovarian cancer | Carboplatin or Paclitaxel | Sensitizes | High PGC-1α subset are more sensitive | (10) |
| Ovarian cancer | Cisplatin | Sensitizes | Resistant cells diminished PGC-1α, although high-grade serous carcinomas had elevated PGC-1α | (11) |
| Acute Myeloid Leukemia | Cytarabine and doxorubicin, and VEGFR2 inhibition | Sensitizes to new treatment | Presence of PGC-1α is required for VEGFR2 inhibition to resensitize cells to chemotherapy | (12) |
| Clear Cell Renal Cell Carcinoma | Doxorubicin or radiation | Sensitizes | Resistant cells respond better with elevated PGC-1α, cells with elevated PGC-1α are more sensitive | (13) |
| Pancreatic TICs | Metformin | Sensitizes | High PGC-1α leads to higher OXPHOS and better response to metformin | (14) |
| Breast cancer | Methotrexate | Sensitizes | Increasing PGC-1α increases sensitivity by diminishing expression of the target pathway | (15) |

**Supplementary References**

1. Yao Z, Jones AW, Fassone E, Sweeney MG, Lebiedzinska M, Suski JM, Wieckowski MR, Tajeddine N, Hargreaves IP, Yasukawa T, Tufo G, Brenner C, Kroemer G, Rahman S, Szabadkai G. PGC-1beta mediates adaptive chemoresistance associated with mitochondrial DNA mutations. Oncogene. 2013;32(20):2592-600. Epub 2012/07/11.

2. Shen L, Sun B, Sheng J, Yu S, Li Y, Xu H, Su J, Sun L. PGC1alpha promotes cisplatin resistance in human ovarian carcinoma cells through upregulation of mitochondrial biogenesis. International journal of oncology. 2018;53(1):404-16. Epub 2018/05/12.

3. Kim B, Jung JW, Jung J, Han Y, Suh DH, Kim HS, Dhanasekaran DN, Song YS. PGC1alpha induced by reactive oxygen species contributes to chemoresistance of ovarian cancer cells. Oncotarget. 2017;8(36):60299-311. Epub 2017/09/28.

4. Andrzejewski S, Klimcakova E, Johnson RM, Tabaries S, Annis MG, McGuirk S, Northey JJ, Chenard V, Sriram U, Papadopoli DJ, Siegel PM, St-Pierre J. PGC-1alpha Promotes Breast Cancer Metastasis and Confers Bioenergetic Flexibility against Metabolic Drugs. Cell metabolism. 2017;26(5):778-87 e5. Epub 2017/10/11.

5. Vellinga TT, Borovski T, de Boer VC, Fatrai S, van Schelven S, Trumpi K, Verheem A, Snoeren N, Emmink BL, Koster J, Rinkes IH, Kranenburg O. SIRT1/PGC1alpha-Dependent Increase in Oxidative Phosphorylation Supports Chemotherapy Resistance of Colon Cancer. Clinical cancer research : an official journal of the American Association for Cancer Research. 2015;21(12):2870-9. Epub 2015/03/18.

6. Park S, Chang CY, Safi R, Liu X, Baldi R, Jasper JS, Anderson GR, Liu T, Rathmell JC, Dewhirst MW, Wood KC, Locasale JW, McDonnell DP. ERRalpha-Regulated Lactate Metabolism Contributes to Resistance to Targeted Therapies in Breast Cancer. Cell reports. 2016;15(2):323-35. Epub 2016/04/07.

7. Vazquez F, Lim JH, Chim H, Bhalla K, Girnun G, Pierce K, Clish CB, Granter SR, Widlund HR, Spiegelman BM, Puigserver P. PGC1alpha expression defines a subset of human melanoma tumors with increased mitochondrial capacity and resistance to oxidative stress. Cancer Cell. 2013;23(3):287-301. Epub 2013/02/19.

8. Ye F, Zhang Y, Liu Y, Yamada K, Tso JL, Menjivar JC, Tian JY, Yong WH, Schaue D, Mischel PS, Cloughesy TF, Nelson SF, Liau LM, McBride W, Tso CL. Protective properties of radio-chemoresistant glioblastoma stem cell clones are associated with metabolic adaptation to reduced glucose dependence. PloS one. 2013;8(11):e80397. Epub 2013/11/22.

9. Gopal YN, Rizos H, Chen G, Deng W, Frederick DT, Cooper ZA, Scolyer RA, Pupo G, Komurov K, Sehgal V, Zhang J, Patel L, Pereira CG, Broom BM, Mills GB, Ram P, Smith PD, Wargo JA, Long GV, Davies MA. Inhibition of mTORC1/2 overcomes resistance to MAPK pathway inhibitors mediated by PGC1alpha and oxidative phosphorylation in melanoma. Cancer Res. 2014;74(23):7037-47. Epub 2014/10/10.

10. Gentric G, Kieffer Y, Mieulet V, Goundiam O, Bonneau C, Nemati F, Hurbain I, Raposo G, Popova T, Stern MH, Lallemand-Breitenbach V, Muller S, Caneque T, Rodriguez R, Vincent-Salomon A, de The H, Rossignol R, Mechta-Grigoriou F. PML-Regulated Mitochondrial Metabolism Enhances Chemosensitivity in Human Ovarian Cancers. Cell metabolism. 2018. Epub 2018/09/25.

11. Gabrielson M, Bjorklund M, Carlson J, Shoshan M. Expression of mitochondrial regulators PGC1alpha and TFAM as putative markers of subtype and chemoresistance in epithelial ovarian carcinoma. PloS one. 2014;9(9):e107109. Epub 2014/09/23.

12. Nobrega-Pereira S, Caiado F, Carvalho T, Matias I, Graca G, Goncalves LG, Silva-Santos B, Norell H, Dias S. VEGFR2-Mediated Reprogramming of Mitochondrial Metabolism Regulates the Sensitivity of Acute Myeloid Leukemia to Chemotherapy. Cancer Res. 2018;78(3):731-41. Epub 2017/12/13.

13. LaGory EL, Wu C, Taniguchi CM, Ding CC, Chi JT, von Eyben R, Scott DA, Richardson AD, Giaccia AJ. Suppression of PGC-1alpha Is Critical for Reprogramming Oxidative Metabolism in Renal Cell Carcinoma. Cell reports. 2015;12(1):116-27. Epub 2015/06/30.

14. Sancho P, Burgos-Ramos E, Tavera A, Bou Kheir T, Jagust P, Schoenhals M, Barneda D, Sellers K, Campos-Olivas R, Grana O, Viera CR, Yuneva M, Sainz B, Jr., Heeschen C. MYC/PGC-1alpha Balance Determines the Metabolic Phenotype and Plasticity of Pancreatic Cancer Stem Cells. Cell metabolism. 2015;22(4):590-605. Epub 2015/09/15.

15. Audet-Walsh E, Papadopoli DJ, Gravel SP, Yee T, Bridon G, Caron M, Bourque G, Giguere V, St-Pierre J. The PGC-1alpha/ERRalpha Axis Represses One-Carbon Metabolism and Promotes Sensitivity to Anti-folate Therapy in Breast Cancer. Cell reports. 2016;14(4):920-31. Epub 2016/01/26.
