# Supplementary material for: Resistance to different anthracycline chemotherapeutics elicits distinct and actionable primary metabolic dependencies in breast cancer
Source: eLife. 2021 Jun 28;10:e65150. doi: 10.7554/eLife.65150 (PMC8238502; doi:10.7554/eLife.65150)
Supplement: Supplementary file 2. [file elife-65150-supp2.docx]

**Supplementary File 2: List of primer sequences for RT-qPCR**

| Gene | Forward primer sequence 5’-3’ | Reverse primer sequence 5’-3’ |
| --- | --- | --- |
| *ABCB1* | GCAGCTGGAAGACAAATACACAA | CCCAACATCGTGCACATCA |
| *ABCC1* | GCTGGAGTGTGTGGGCAACT | CTGAGGCTGTGCCTGGAGAT |
| *AKR1C3* | TCTAAAGCCAGGTGAGGAAC | CAGAGCACTATAGGCAACCA |
| *CAT* | gacaatcagggtggtgctcc | gaatcgcattcttaggcttctc |
| *CS* | CAACTCAGGACGGGTTGTTCCAGG | GTAGTAATTCATCTCCGTCATGCC |
| *FTH1* | AGTGCTTGGACGGAACC | ATCGCGGTCAAAGTAGTAAGA |
| *GCLC* | GGACAAACCCAAACCATCCT | TGAACCCAGGACAGCCTAAT |
| *GCLM* | TGAATGGAGTTCCCAAATCAAC | GAGATACAGTGCATTCCAAGAC |
| *GLS* | GGTCTCCTCCTCTGGATAAGATGG | CCCGTTGTCAGAATCTCCTTGAGG |
| *GLS2* | GGCAGAGAGACGCCACACAG | AGTGGCCTTTAGTGCAGTGGT |
| *GLUL* | CTCGCGGCCTAGCTTTACCC | CCACTCAGGCAACTCTTCCACA |
| *GOT1* | ACTGCCGACTTCAGGGAGGA | GCACACCTCCTACCCGCTTC |
| *GOT2* | CTGGGAGTTGGTGCCTACCG | CTGCACAGTGACAAACCGGC |
| *GPX3* | GGGGACAAGAGAAGTCGAA | AGGGCTCCGTACTCGTAAAT |
| *GSR* | TGATTCAATGATCAGCACCAAC | CAGTAACCATGCTGACTTCCA |
| *GSS* | CCTGGCTGAGGGAGTATTG | TTTGATGGTGCTGGAAAGAGT |
| *HMOX2* | GACAGGCGACAGCGAC | GGTCAGCCATTCTCATTTGG |
| *IDH1* | ACCAATCCCATTGCTTCCATTTTT | TCAAGTTTTCTCCAAGTTTATCCA |
| *IDH2* | CAGGAGATCTTTGACAAGCAC | ATGAGGTCTTGGTTCCCATC |
| *LDHA* | ATTCCCGATTCCTTTTGGTTC | GCAAGTTCATCTGCCAAGTC |
| *MDH2* | GCTCTGCCACCCTCTCCATG | TTTGCCGATGCCCAGGTTCTTCTC |
| *ME1* | GGAACCCTCACCTCAACAA | GAGAAGATACCTGTCAAAGTCAG |
| *ME2* | TACACTTACAGAGGGCAGGT | GCCGGGTGTTACAGAGAATA |
| *NFE2L1* | CCAGACAAGTGGGAGTGAAA | ATTCAAAGTGGGGAAAAAGTGC |
| *NFE2L2* | CGCAATTGCTATTTTCCCCA | GCCGAAGAAACCTCATTGTC |
| *NQO1* | GGACATCACAGGTAAACTGAAG | TTTCAGAATGGCAGGGACTC |
| *PC* | GGAGAGCTGACCAAACACTAA | ATGGCAATCTCACCTCTGTTG |
| *PPARGC1A* | GTGCTGCTCTGGTTGGTGAAGA | CACCACTTGAGTCCACCCAGAAAG |
| *PPARGC1B* | GTACATTCAAAATCTCTCCAGCGACATG | GAGGGCTCGTTGCGCTTCCTCAGGGCAG |
| *PRDX3* | cctttggatttcacctttgtgtg | caaaccaccattctttcttggtg |
| *PRDX5* | ccaatcaaggtgggagatgcc | gcaggtgtgtcttggaacatc |
| *SLC1A5* | CAAGGAGGTGCTCGATTCGT | ACCCTGGTTCCGGTGATATTC |
| *SOD2* | GCTGCTCTATTGTAGCATTTCT | CATCCCTACAAGTCCCCAAA |
| *TXNRD2* | gtccacaccactgtgcgtgg | ttgcagggagatggctcagcg |
