# Supplementary material for: Resistance to different anthracycline chemotherapeutics elicits distinct and actionable primary metabolic dependencies in breast cancer
Source: eLife. 2021 Jun 28;10:e65150. doi: 10.7554/eLife.65150 (PMC8238502; doi:10.7554/eLife.65150)
Supplement: Supplementary file 3. [file elife-65150-supp3.docx]

**Supplementary File 3: List of primer sequences for ChIP**

| Gene | Forward primer sequence 5’-3’ | Reverse primer sequence 5’-3’ |
| --- | --- | --- |
| *ABCB1* | AGCTGAAAGCAGCAGGGAAGAGG | ACCTGGAGTGTTTACAGTTCCCCT |
| *ABCC1* | CCTCCTTCCCTCGCTAGGTCC | GGCGAGGCCGCAGAGTGTA |
| *AKR1C3* | GGGCGCACGGCAAGCTATTC | AGCACACACACCACTCCCTGT |
| *CAT* | GGTCAAGCCCAGGTGCAACAT | TCCTGCAAGGCCTGGGTCAAC |
| *FTH1* | AGGCTGGCTGGGAACAATGGAA | AAAGGTCCCAAGACCAGGCCAC |
| *GSR* | GCAGGAACCCAGGACCGCAA | TGCAAAGATCGCCCAAAGTCAAGG |
| *GSS* | CCGACACCGGCCCGATCTAA | ATTGGAATTCCGGAGGCCGGG |
| *HMOX2* | TCAGCTACAAGGGGCGTGTCA | AAGGAAGGCCCCGGGAGGG |
| *NFE2L2* | ACACTCGCAACTCTTACCCTTGACA | ATAGCGTGCAAACCTCGCCG |
| *NQO1* | GCGGGACCCAACGCCTGAAT | CAGTTGCTCCGGCGGGTGAG |
| *PC* | AAGGAGAAGGCGCTTGTGCCA | GCTTCCAAACCCCCTAGGTCCTG |
| *PC_2* | ATGTGCCCAAGGTCACAAGGCT | CAGCGCTGCCCCTAATGTCC |
| *Neg_1* | GAAGCCCATTTTCCCTCCCA | CAATGGGGCTGAAGCACAAC |
| *Neg_2* | CTGGTCTCACCTACCTTCCTGT | ATCCATGAACTCCAGGAGCTCA |
